# The Trypanosome Rab-Related Proteins RabX1 and RabX2 Play No Role in IntraCellular Trafficking but May Be Involved in Fly Infectivity

**DOI:** 10.1371/journal.pone.0007217

**Published:** 2009-09-29

**Authors:** Senthil Kumar A. Natesan, Lori Peacock, Ka Fai Leung, Keith R. Matthews, Wendy Gibson, Mark C. Field

**Affiliations:** 1 Department of Pathology, University of Cambridge, Cambridge, United Kingdom; 2 School of Biological Sciences, University of Bristol, Bristol, United Kingdom; 3 Institute of Immunology and Infection Research, University of Edinburgh, Edinburgh, United Kingdom; Katholieke Universiteit Leuven, Belgium

## Abstract

**Background:**

Rab GTPases constitute the largest subgroup of the Ras superfamily and are primarily involved in vesicle targeting. The full extent of Rab family function is unexplored. Several divergent Rab-like proteins are known but few have been characterized. In *Trypanosoma brucei* there are sixteen Rab genes, but RabX1, RabX2 and RabX3 are divergent within canonical sequence regions. Where known, trypanosome Rab functions are broadly conserved when orthologous relationships may be robustly established, but specific functions for RabX1, X2 and X3 have yet to be determined. RabX1 and RabX2 originated *via* tandem duplication and subcellular localization places RabX1 at the endoplasmic reticulum, while RabX2 is at the Golgi complex, suggesting distinct functions. We wished to determine whether RabX1 and RabX2 are involved in vesicle transport or other cellular processes.

**Methodology/Principal Findings:**

Using comparative genomics we find that RabX1 and RabX2 are restricted to trypanosomatids. Gene knockout indicates that RabX1 and RabX2 are non-essential. Simultaneous RNAi knockdown of both RabX1 and RabX2, while partial, was also non-lethal and may suggest non-redundant function, consistent with the distinct locations of the proteins. Analysis of the knockout cell lines unexpectedly failed to uncover a defect in exocytosis, endocytosis or in the morphology or location of multiple markers for the endomembrane system, suggesting that neither RabX1 nor RabX2 has a major role in intracellular transport. However, it was apparent that RabX1 and RabX2 knockout cells displayed somewhat enhanced survival within flies.

**Conclusions/Significance:**

RabX1 and RabX2, two members of the trypanosome Rab subfamily, were shown to have no major detectable role in intracellular transport, despite the localization of each gene product to highly specific endomembrane compartments. These data extend the functional scope of Rab proteins in trypanosomes to include non-canonical roles in differentiation-associated processes in protozoa.

## Introduction

*Trypanosoma brucei* is the protozoan parasite causing African sleeping sickness in humans and a similar disease in sylvatic and domestic animals [1]. *T. brucei* is also a member of the Excavata eukaryotic supergroup, which is highly distant from animals and fungi, and represents an important model organism for evolutionary cell biology. The life cycle consists of numerous developmental steps and includes two experimentally tractable proliferative stages, the bloodstream form (BSF) in mammals and the procyclic culture form (PCF) in the tsetse fly [2]. Trypanosomes grow as proliferative slender BSFs in the mammalian host, changing to non-proliferative stumpy forms by a density-sensing mechanism as parasite numbers increase [3], [4]. Stumpy forms are cell-cycle arrested and attain a state of commitment where they cannot revert to long slender forms [5], but instead can differentiate on to PCFs and re-enter the cell cycle if taken up in a bloodmeal by the tsetse fly vector [6]. PCFs first establish an infection in the midgut, from where they progress anteriorly to the salivary glands and differentiate to infective forms [7]. In the laboratory, *T. b. brucei* strain Lister 427 is extensively used to study trypanosome biology, but most laboratory-derived lines of this strain are unable to complete development in the fly [8], [9], [10].

Trypanosomes undergo major changes in their membrane trafficking system, surface antigen expression, cell structure and metabolism during the transition from mammalian to insect host, and the differentiation program from BSF to PCF has been well-studied [5], [11]. For example, endocytosis is developmentally regulated and ∼ten-fold up-regulated in BSFs compared with PCFs [12]. Increased endocytosis in the BSF potentially offers protection against the mammalian immune system by efficient recycling of the variant surface glycoprotein (VSG) coat and rapid capping and internalization of anti-VSG antibodies [13], [14], [15]. The endocytic apparatus and its components have been well described for *T. brucei* [16], [17], including several small Ras-like GTPases [18], [19].

The Ras superfamily includes Ras, Rho and Cdc42, which primarily mediate signal transduction. The Rho, Cdc42 and Rac families also participate in cytoskeletal activities, while Rab, Ran and Arf (ADP-ribosylation factor) proteins are involved mainly in transport processes [19]. These molecules act as molecular switches and also as sites for assembly of supramolecular complexes, making the precise delineation of function more challenging. However, essentially all GTPases act as signal transducers at some level by virtue of differential protein-protein interaction based on the nucleotide state, specifically GTP versus GDP. The Rab proteins are predominantly membrane-bound and play a major role in secretory and endocytic pathways. By interaction with SNAREs, cytoskeletal elements and other factors, Rabs facilitate the process of vesicle targeting, docking and budding, as well as providing an important component of the specificity module [20].

The *T. brucei* genome encodes over 40 small GTPases, of which at least 30, or 75%, are Rab and Arf family members and hence predicted to be involved in vesicle transport. The remaining Ras superfamily proteins are probably involved primarily in signal transduction and cytoskeletal functions [8], [21]. The trypanosome Rab family consists of 16 members, many of which are known to participate in endocytic and exocytic pathways. A core set have clear orthologues in yeast and mammals and are highly conserved across evolution [11], [18], [22]. These include Rab1 and Rab2 [ER/Golgi transport, [23]], Rab5A and Rab5B [early endosomes, [15]], Rab4 and Rab11 [recycling, [24], [25]], Rab7 [delivery to late endosomes, [26]] and Rab6 [retrograde transport through the Golgi, [27]]. The *T. brucei* Rab family also includes Rab18, Rab21, Rab23 and Rab28 that are orthologous to mammalian Rab proteins though absent from *Saccharomyces cerevisiae* [18], and which are expected to have broadly similar functions to their higher eukaryote orthologues. However, much of the overall view of the functions of less well conserved GTPases in trypanosomes is reliant on *in silico* prediction and is unsupported by direct experimental analysis. Given the evolutionary divergence of trypanosomes and higher eukaryotes, direct empirical evidence is essential for establishment of function.

The *T. brucei* Rab family also includes three divergent Rabs, RabX1, RabX2 and RabX3. The protein products of these genes are predicted to lack the full complement of Rab canonical sequence motifs [19]. In *T. brucei*, RabX1 (previously TbRab2A/Trab1) and RabX2 (previously TbRab31/Trab7) were identified as a pair of Rab genes that are estimated to have arisen as a result of a duplication ∼100 million years ago, followed by sequence divergence and acquisition of distinct functions [28]. RabX1 localizes to the endoplasmic reticulum (ER) in *T. brucei* and to the ER-Golgi intermediate compartment (ERGIC) when expressed in mammalian cells [23]. RabX1 is membrane bound, expressed at similar levels in both BSF and PCF stages and throughout the cell cycle [28]. Over-expression of RabX1 in PCF parasites results in down-regulation of procyclin biosynthesis but up-regulation of total protein biosynthesis in addition to accumulation of ER-derived vesicular structures, suggesting a role in ER function [23]. The RabX2 gene is located immediately adjacent to RabX1 on chromosome VIII [28]. The RabX2 protein is localized to the Golgi complex but shows variation in the pattern of localization between BSFs and PCFs, which may reflect the underlying architecture of the Golgi apparatus [29]. Therefore, although the cellular locations of both RabX1 and RabX2 are well established, their definitive functions remain unclear with few clues about possible roles. Firstly, the core set of Rab proteins involved in the major steps of vesicle trafficking in trypanosomes has been identified, leaving no obvious niche for RabX1 and RabX2. Secondly, *in silico* analysis shows that, while the core set of Rab proteins involved in vesicle trafficking have clear orthologues in higher eukaryotes, RabX1 and RabX2 are so divergent that inference from other systems is not possible. Thus potentially RabX1 and RabX2 may have unique roles in trypanosomes. Here we addressed the functions of RabX1 and RabX2 by generating double gene knockout cell lines. Our results suggest a potential role for both RabX1 and RabX2 in fly infectivity and possibly differentiation from BSF to PCF but not major participation in vesicle trafficking.

## Materials and Methods

### Ethics statement

All cell lines for the present work were generated in house. Genetic modification to and containment of trypanosomes was authorized by University of Cambridge Biological Safety and Ethics review panel and University of Bristol Biological and Genetic Modification Safety Committee. Animal (mouse) experiments complied with local ethical rules and UK Home Office license regulations relevant to work carried out at the University of Edinburgh.

### Trypanosomes, in vitro culture and mouse infections

Bloodstream form *Trypanosoma brucei brucei* MITat 1.2 (M221 strain) and procyclic form *T. b. brucei* MITat 1.2 (Lister 427) were grown at 37°C in HMI-9 or at 27°C in SDM-79 respectively with supplements as previously described [30], [31]. RNAi experiments were performed using the Single Marker Bloodstream form (SMB) or the tetracycline-based PTT procyclic line as described previously. To estimate their ability to infect mice *in vivo*, 10,000 wild type and Rab X1 and RabX2 knockout BSF parasites were inoculated into groups of five age, sex and weight matched MF1 mice pre-treated with cyclophosphamide. In each case, the level of parasitaemia was determined by tail bleed and counting parasites under a microscope over a period of 2 to 6 days post-infection using the rapid matching method described previously [32]. Humane end-points for mouse infections were followed according to UK Home Office requirements.

### Tsetse fly infections

Male and female tsetse flies from the Bristol laboratory colony of *Glossina morsitans morsitans* were caged in groups of 25, maintained at 25°C and 70% relative humidity, and fed on sterile defibrinated horse blood *via* a silicone membrane. Flies were given an infected bloodmeal for their first feed 24–48 hours post-eclosion containing ∼10^6^ cultured BSF trypanosomes per ml, supplemented with 10 mM L-glutathione [33] to increase infection rates. Flies were dissected in PBS 4–5 days after infection and whole tsetse alimentary tracts from the proventriculus to the rectum were viewed as wet mounts under bright field illumination (×100 magnification). The relative number of trypanosomes was scored on a 5-point scale: none, negligible (≤5 trypanosomes), low (few trypanosomes scattered in midgut), moderate (few trypanosomes scattered in different areas of midgut), high (trypanosomes throughout midgut). Results from male and female tsetse were pooled and a chi-squared test used for comparison of infection levels between cell lines.

### Recombinant DNA manipulations

RabX1 RNAi in BSF was performed using a recombinant p2T7 plasmid containing a 440 bp long fragment of RabX1 [23]. The RabX2 open reading frame (ORF) sequence was obtained from GeneDB, and analyzed using RNAit; a 250 bp region that would ensure specificity for down-regulation was identified [34] and was PCR amplified from BSF genomic DNA with Taq DNA polymerase using primers X2RNAi-F (CAAGCTTAGGGATTTGCAGG) and X2RNAi-R (TCAGCACCTCCACTTCCTCT), and cloned into p2T7^TAblue^ using the Eam1105I sites. To generate RNAi constructs for PCFs p2T7-177 was used where the RabX1 and RabX2 RNAi DNA fragments were excised from their respective p2T7^TAblue^ constructs using HindIII and XhoI and ligated into BamHI- and XhoI-digested p2T7-177. p2T7^TAblue^-RNAi and p2T7-177-RNAi plasmids were digested with NotI or BstXI respectively before transfection into BSF SMB or PCF PTT lines.

A p2T7-RabX1-RabX2 double RNAi construct was generated first by PCR amplification of the RabX1 insert with Taq DNA polymerase, using RabX1dKD-F (TTTTCGAATGCACCAAATGA) and RabX1dKD-R (CGGAGGTATCCCAAATCTGAGAATTCACTTGGGGACAGACCCTTTC), and the RabX2 insert, using RabX2dKD-F (GAAAGGGTCTGTCCCCAAGTGAATTCTCAGATTTGGGATACCTCCG) and RabX2dKD-R (TCAGCACCTCCACTTCCTCT). 10 ng of each PCR insert, which contains an overlapping region, were used as PCR template to amplify the double RNAi insert using primers RabX1dKD-F and RabX2dKD-R. The double RNAi insert was cloned into p2T7^TAblue^ using Eam1105I sites as above.

To generate the double gene knockout constructs ∼1 kb DNA fragments from the 5′ UTRs of RabX1 and RabX2 were PCR amplified using primers RabX1 5′UTR-F (GTGGTACCGAAAGGACAGGGAACGGAAG), RabX1 5′UTR-R (GCCTCGAGATGCGAAGTGGACTCTTCAA) and RabX2 5′UTR-F (GCGGTACCTCCAATGTGTCGTGAAGTCT), RabX2 5′UTR-R (GCTCGAGGTAAGGAAACTTCACCTAACCC). The PCR products were digested with KpnI and XhoI and cloned into pXS5:NEO to generate pXS5-X15′UTR:NEO and pXS5-X25′UTR:NEO. ∼1 kb DNA fragments from the 3′ UTRs of RabX1 and RabX2 were PCR amplified using the primers RabX1 3′UTR-F (GTGATCTTCGTTCGTACTAGTGAGAGG), RabX1 3′UTR-R (GTGAGCTCGGTGCAACAAACTACTACT) and RabX2 3′UTR-F (GCACTAGTACCTCTTATGGTGGCAATAGCA), RabX2 3′UTR-R (GTGAGCTCATGCGAAGTGGACTCTTCAAAC) respectively. PCR-amplified 3′ UTR of RabX1 and RabX2 were digested with SpeI and SacI and cloned into pXS5 -X1-5′UTR:NEO and pXS5-X25′UTR:NEO to generate pXS5-X15′&3′UTR:NEO and pXS5-X25′&3′UTR:NEO respectively. Both pXS5-X15′&3′UTR:NEO and pXS5-X25′&3′UTR:NEO were used to replace the first copy of RabX1 and RabX2 in the genome. To target the second copy of the genome the neomycin coding gene in the pXS5-X15′&3′UTR:NEO and pXS5-X25′&3′UTR:NEO was removed by digestion with *Asc*I and *Pac*I and replaced by a DNA fragment encoding for hygromycin-resistance generated by PCR from pXS5:HYG, using primers Hygro-F (GTGGCGCCATGAAAAAGCCTGAACTCAC) and Hygro-R (GCTTAATTAATTCCTTTGCCCTCGGACGAGTGC) and resulting in pXS5-X15′&3′UTR:HYG and pXS5-X25′&3′UTR:HYG.

To generate add-back versions of RabX1 and RabX2, the plasmid pHD1034 was used. pHD1034, contains a puromycin resistance gene and a ribosomal RNA promoter and is inserted into the ribosomal spacer of *T. brucei* for constitutive expression in all life stages [35]. The RabX1 ORF was obtained from pJBKT7RabX1WT (Gabernet-Castello and MCF, unpublished) after digestion with EcoRI and BamHI. RabX2 ORF was PCR amplified from genomic DNA using the primers RabX2-F (TACGAAGCTTATGAAAGAGGAACCC) and RabX2-R (GCGGATCCTCAGCACCTCCACTTCCT). RabX1 and RabX2 ORFs were then cloned into pHD1034 using the restriction sites HindIII and BamHI. pHD1034-RABX1 or pHD1034-RABX2 was digested with NotI before transfecting with RabX1 and RabX2 2KO (gene knockout) parasites. All constructs were sequence verified prior to use in transfections.

### Quantitative real-time PCR

Total RNA from *T. brucei* BSF cells was extracted using the Qiagen RNeasy mini kit and total RNA from different life stages of *T. cruzi* and *L. mexicana* were gifts. Synthesis of cDNA was performed in a 25 µl reaction volume with 2 µg RNA and oligo dT primers using the Superscript II Reverse Transcriptase kit (Invitrogen). Further, PCR amplification was performed either under standard PCR conditions or in a reaction mixture containing cDNA and iQ SYBR Green supermix using a MiniOpticon Instrument (BioRad). RabX1 and RabX2 from *L. mexicana* were amplified using *L. mexicana* RabX1-F (CATTGGTGACAGTGGCGTAG), *L. mexicana* RabX1-R (ACTTGTTCTCCTTGCGGTTG) and *L. mexicana* RabX2-F (ATCGTGATTGGGAGCGTTAG), *L. mexicana* RabX2-R (GGTAGCAGAGGCAGCTATGG) respectively. RabX1 and RabX2 from *T. cruzi* were amplified using *T. cruzi* RabX1-F (CTCTGACGGTGCGTCTATCA), *T. cruzi* RabX1-R (GCTTTCTGACCTTCCTGCAC) and *T. cruzi* RabX2-F (TGTGACGGGACATTTTACGA), *T. cruzi* RabX2-R (CCCCTCGATCTCACGATTTA) respectively. RabX1 and RabX2 from *T. brucei* were amplified using *T. brucei* RabX1-F (AGGATTACGCATCCACCATC), *T. brucei* RabX1-R (GCAGTTGCCACTGACTGAAA) and *T. brucei* RabX2-F (GGGTGTAGGGAAGAGCAACA), *T. brucei* RabX2-R (GCGCGTGCTTTTCTAAAGTT) respectively.

### Protein electrophoresis and Western blotting

SDS lysates from 1×10^6^–1×10^7^ cells were separated on 12% SDS-polyacrylamide gels and wet-blotted onto PVDF membrane (Immobilon, Millipore, Bedford, MA), blocked with 5% milk in TBS-T (Tris-buffered saline, 0.5% Tween 20) for two hours at room temperature and probed with antibody to RabX1 at 1∶1000, RabX2 at 1∶1000, BiP (gift from J. Bangs) at 1∶10 000, PAD1 at 1∶1000 and Procyclin (Cedarlane Laboratories Ltd) at 1∶5000 in 1% milk followed by HRP-conjugated goat anti-rabbit IgG (Sigma) or rabbit anti-mouse IgG (Sigma) at 1∶10 000 dilution in 1% milk in TBS-T. Detection was by chemiluminescence and exposure to X-ray film (Kodak BioMax MR).

### Southern Blotting

Southern blotting was performed using 5 µg of genomic DNA isolated from BSF or PCF parasites in log phase [36]. To release the RabX1 or RabX2 locus from the genome, DNA was digested with PstI and SacII. Further, the digested DNA was separated by electrophoresis and transferred to a nitrocellulose membrane and probed with specific probes for RabX1 and RabX2. A 255 bp DNA fragment was PCR amplified using primers X1probe-F (ATGATCACAGCAGCTTCCCC) and X1-probe-R (GCGGGTAAAAGGCAGTTGCCAC) to generate RabX1probe. A 170 bp DNA fragment was PCR amplified using primers X2probe-F (GAGACGAGCTCGAGAAATAACAC) and X2RNAi-R to generate RabX2probe. Hybridization and washing was done as described previously [37].

### Immunofluorescence

Trypanosomes were harvested by centrifugation, washed with PBS and fixed with 4% PFA in ice-cold vPBS. Immunofluorescence was performed as described previously [38] with modifications. Staining was as described [38], with primary antibody concentrations of anti-RabX1 at 1∶200, anti-RabX2 at 1∶200, anti-CLH at 1∶1000, anti-ISG65 at 1∶1000, anti-p67 at 1∶500, anti-Rab1 at 1∶200, anti-Rab2 at 1∶200, and anti-BiP at 1∶1000 and secondary antibodies anti-rabbit Cy3 (Sigma) at 1∶1000 and anti-mouse Oregon Green (Molecular Probes) at 1∶1000. Specimens were analyzed on a Nikon Eclipse epifluorescence microscope equipped with a Hamamatsu CCD camera and data collected in Metamorph under non-saturating conditions (Molecular Devices). For presentation only, acquired gray scale images were false-colored, enhanced and assembled in Adobe Photoshop CS (Adobe Systems Inc); quantitative analysis was performed on the raw data.

### Electron microscopy

BSF parasites grown to logarithmic phase were harvested and washed 3 times with excess amounts of wash buffer (0.9% NaCl, 0.1 M HEPES, pH 7.0). Washed cells were fixed with 4% PFA and processed for EM as described [39].

*VSG export assay:* VSG export was performed as described previously [40] with a few modifications. BSF parasites grown to logarithmic phase were harvested and washed with labeling medium (Met/Cys-free RPMI-1640 medium supplemented with dialyzed FCS) pre-warmed to 37°C. Parasites were starved by incubation with 1 ml of labeling medium for 15 minutes at 37°C. Parasites were then pulse labeled with ^35^S-Promix (Amersham) to a final concentration of 200 µCi/ml and incubated for 7 minutes at 37°C, then diluted 1∶10 with pre-warmed complete HMI-9 and chased for up to 1 hour at 37°C. Aliquots were taken at the desired time intervals and placed on ice to stop exocytosis. Further, parasites were washed with 1 ml of ice-cold PBS/1 mg/ml BSA, and resuspended in 920 µl of hypotonic lysis buffer (10 mM Tris-HCl, pH 7.5) and the parasites were lysed on ice for 5 minutes. The lysates were incubated for 10 minutes at 37°C to enable the endogenous GPI-specific phospholipase C (GPI-PLC) to convert the membrane-form (mf)VSG to soluble (s)VSG. Lysates were centrifuged at 20 000 g for 10 minutes at 4°C to separate the mfVSG (pellet) and sVSG (supernatant). The sVSG fraction was removed and added to 90 µl of 10× buffer (500 mM Tris-HCl pH 7.5, 1.5 M NaCl, 10% NP-40). The mfVSG fraction was resuspended in 1 ml of ice-cold hypotonic lysis buffer and incubated on ice for 25 minutes to lyse the membranes. Both fractions were centrifuged at 20 000 g for 10 minutes at 4°C and to the supernatant was added MnCl_2_ and CaCl_2_ to a final concentration of 1 mM to aid binding of glycoproteins to ConA. VSG was recovered from the samples by incubation with ConA-sepharose 4B for one hour at 4°C. Samples were washed and resuspended in sample buffer and loaded onto 12% SDS-polyacrylamide gels at 10^6^ cell equivalents per lane. Gels were stained, fixed and exposed to X-ray film (Kodak BioMax MR). Image intensity was quantified using NIH ImageJ.

### Transferrin uptake

Mid-log phase BSF cells from culture were harvested and washed in serum-free HMI-9 containing 1% BSA. Cells were resuspended at a concentration of ∼1×10^7^ cells/ml and incubated for 30 minutes at 37°C. To these parasites, 125 µg/ml of Alexa-conjugated transferrin (Molecular Probes) was added and at different time intervals, aliquots were removed and placed immediately on ice. Cells were washed with ice-cold PBS, fixed with 1% formalin and analyzed using a Cyan ADP FACS machine (Dako, Denmark) and the results were analyzed using Summit V4.3 (Dako, Denmark).

In vitro *differentiation:* BSF wild-type or knockout cell lines were harvested at mid-log phase, washed in PBS and then resuspended at a cell density of 5×10^6^ cells/ml in SDM-79 supplemented with 10% FCS, 6 mM citrate, 6 mM *cis*-aconitate and incubated at 27°C.

## Results

### RabX1 and RabX2 are restricted to the Kinetoplastida and expressed in major life stages

**Figure 1:**
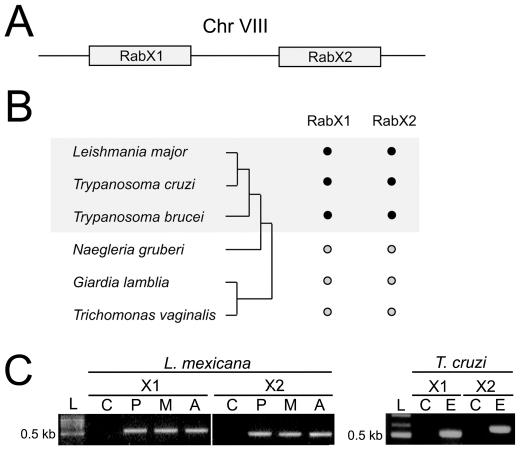
RabX1 and RabX2 are specific to trypanosomatids. (A) Schematic representation of the RabX1 and RabX2 locus arranged on *Trypanosoma brucei* chromosome VIII. (B) Presence of RabX1 and RabX2 encoding sequences from trypanosomes in relation to additional fully sequenced Excavata lineages. RabX1 and RabX2 are found only in trypanosomatids, but not *Naegleria gruberi*, *Giardia lamblia* and *Trichomonas vaginalis*. Closed dots indicate gene is found, open dots not found. Schematic phylogenetic tree to indicate relationships between the lineages is on the left. (C) RT-PCR showing the presence of RabX1 and RabX2 in the major life stages of *L. mexicana* and *T. cruzi.* L, DNA ladder; C, no reverse transcriptase negative control; P, promastigote; M, metacyclic; A, amastigote; E, epimastigote.

RabX1 and RabX2 are adjacent ORFs on chromosome VIII of *T. brucei* (Figure 1A). Comparative genomic analysis using BLAST and phylogenetic reconstruction of RabX1 and RabX2 among fully sequenced representative Excavata protists revealed that these two genes are specific to the Kinetoplastida and not found in non-trypanosomatid lineages (Figure 1B and data not shown). In addition RabX1 and RabX2 ORFs maintain their syntenic and adjacent position in the *T. cruzi* and *L. mexicana* genomes (data not shown).

We investigated expression of RabX1 and RabX2 in *L. mexicana* and *T. cruzi* by performing RT-PCR with total RNA from several life stages. Total RNA from the promastigote, metacyclic and amastigote stages of *L. mexicana* were used to prepare cDNA. PCR amplification of fragments using primers specific for *L. mexicana* RabX1 or RabX2 resulted in the generation of a ∼587 bp (RabX1) or ∼463 bp (RabX2) product in all of the life stages analyzed, corresponding to the expected size (Figure 1C). Total RNA from the epimastigote stage of *T. cruzi* was used to prepare cDNA and PCR amplification using primers specific for *T. cruzi* RabX1 or RabX2 resulted in the generation of a ∼515 bp (RabX1) or ∼561 bp (RabX2) fragments respectively, again of the expected sizes (Figure 1C). Expression of RabX1 and RabX2 in *T. brucei* has been documented previously [29], [41]. These data indicate that RabX1 and RabX2 are conserved within, and restricted to, the trypanosomatids, expressedin major life stages of the life cycles of two species and expressed in a third lineage.

### RNAi-mediated down-regulation of RabX1 and RabX2 has no significant growth defect

**Figure 2:**
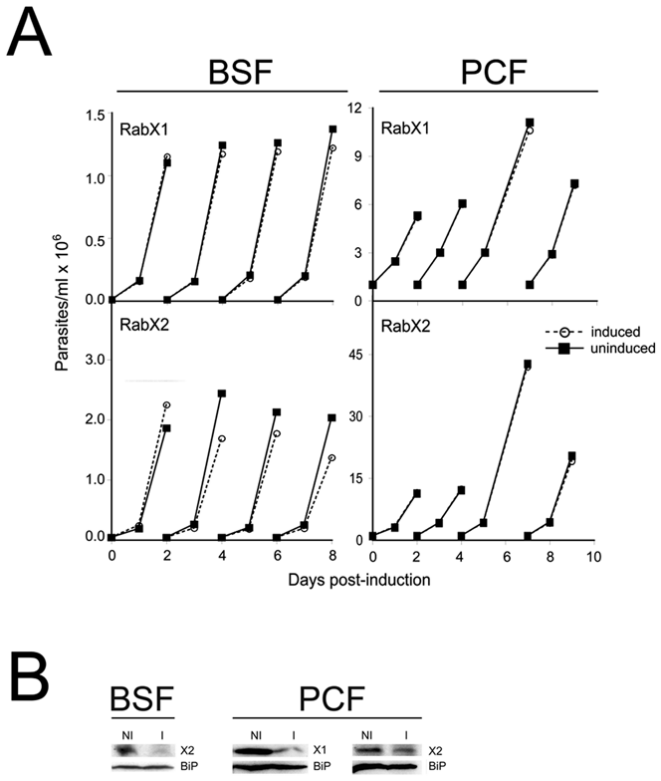
RNAi indicates that RabX1 and RabX2 are non-essential for normal growth of *T. brucei.* Growth curves of BSF SMB and PCF PTT parasites after tetracycline-induced RNAi for RabX1 and RabX2. Top left panel, Growth curve for BSF SMB parasites transfected with p2T7^TAblue^-RabX1 RNAi construct. Bottom left panel, growth curve for BSF SMB parasites transfected with p2T7^TAblue^-RabX2. Top right panel, growth curve for PCF PTT parasites transfected with p2T7-177-RabX1. Bottom right panel, growth curve for PCF PTT parasites transfected with p2T7-177-RabX2. RNAi was induced in BSF SMB parasites by the addition of 1 µg/ml and in PCF PTT lines by the addition of 10 µg/ml of tetracycline. BSF or PCF cells were cultured in the absence (open symbol) or presence (closed symbol) of tetracycline. Insets in the respective growth curves show Western blots demonstrating the RNAi-mediated down regulation of RabX1 or RabX2 along with BiP, an ER marker, as a loading control. Western blot for RabX1 and RabX2 was performed with 1×10^7^ cells after two days of incubation with 1 µg/ml tetracycline in BSF and four days of incubation with 10 µg/ml tetracycline in PCF. NI, non-induced; I, induced. The experiments have been repeated at least twice.

To investigate if RabX1 and RabX2 are essential proteins, we generated RNAi cell lines in both BSF and PCF stages. In BSF, RabX1 and RabX2 RNAi lines were induced with tetracycline to express the respective double-stranded RNA and parasite replication monitored until eight days post-induction. There was no significant growth defect in either the RabX1 and RabX2 RNAi lines when compared to the non-induced parasites (Figure 2). The level of RabX1 protein has been previously reported to be down-regulated by >95% by RNAi-mediated suppression [23]. A similar down-regulation was observed for RabX2 protein after two days of RNAi-mediated suppression (Figure 2). Similarly, RNAi-mediated down-regulation of RabX1 and RabX2 in PCF parasites resulted in no significant growth defect over nine days post-induction. The levels of RabX1 and RabX2 proteins were down-regulated by >80% and ∼60% respectively after four days post-induction demonstrating efficient suppression (Figure 2). Overall these data suggest that neither RabX1 nor RabX2 gene products are required for normal proliferation in BSF and PCF parasites.

### Generation of individual gene knockouts for RabX1 and RabX2 in trypanosomes

**Figure 3:**
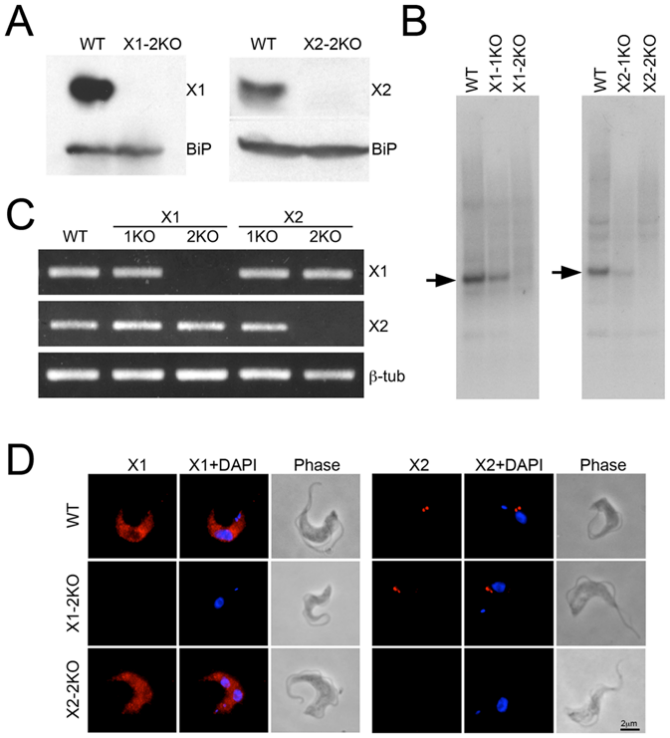
Generation of RabX1-2KO and RabX2-2KO lines. (A) Western blots using 1×10^7^ cells demonstrating the absence of RabX1 and RabX2 protein in their respective 2KO mutants but the ER marker BiP is equally present in wild type and the 2KO mutants. (B) Southern blots using 5 µg of genomic DNA shows the absence of RabX1 and RabX2 genes in their respective 2KO mutants, while the 1KO mutants show reduced amounts of RabX1 and RabX2 hybridization compared to wild type DNA. (C) RT-PCR using RNA obtained from wild type, 1KO and 2KO mutants indicates the absence of RabX1 RNA in X1-2KO and RabX2 RNA in X2-2KO, while the levels of beta-tubulin are unaltered between the wild type, 1KOs and 2KOs. (D) Immunofluoresence indicating absence of RabX1 in X1-2KO and RabX2 in X2-2KO. Parasites were stained with affinity-purified antibody to RabX1 or RabX2 (red) and cells were counterstained with DAPI (blue) for DNA. Phase-contrast images are shown next to the respective fluorescence images. Scale bar 2 µm.

To investigate the functions of RabX1 and RabX2 more fully we generated independent gene knockout cells in BSFs. Gene knockout cells were generated by sequentially replacing each copy of the RabX1 or RabX2 genes by homologous recombination using first a construct containing the neomycin resistance gene plus ∼1 kb sequences from the 5′ and 3′ UTRs of the respective genes, followed by a construct containing the hygromycin-resistance marker with ∼1 kb sequences from the 5′ and 3′ UTRs of the respective genes. Clones with knockout for both alleles of each gene were selected by culturing transfected cells with hygromycin and neomycin and initially confirmed by immunoblotting (Figure 3A). Western blotting confirmed that the RabX1 protein was not detectable in RabX1-2KO cells and that RabX2 protein was similarly undetectable in RabX2-2KO cells (Figure 3A). To confirm the deletion of both copies of the respective genes in the complete allele ockouts, we performed Southern blotting. Digestion of wild type genomic DNA with enzymes PstI and SacII results in a ∼2.2 kb DNA fragment which includes the complete RabX2 ORF and a part of the RabX1 ORF. Using specific probes targeting RabX1 or RabX2 ORF regions in the excised DNA fragment, we were able to confirm the complete deletion of RabX1 or RabX2 coding regions in their respective knockout mutants (Figure 3B). In the single knockout mutants we detected a ∼50% loss of hybridization signal from DNA fragments encoding the respective genes, but there was no detectable hybridization in the knockout cells where both alleles had been removed (Figure 3B). Results from Southern blotting indicate that both alleles of RabX1 and RabX2 have been deleted from the genome in their respective knockout cells.

We performed RT-PCR with RNA extracted from knockout cells to confirm the absence of RabX1 or RabX2 mRNAs. RT-PCR confirmed the absence of RabX1 mRNA in RabX1-2KO and the absence of RabX2 mRNA in RabX2-2KO (Figure 3C). As RabX1 and RabX2 are adjacent in the genome we also analyzed the expression of RabX2 in RabX1-2KO and the expression of RabX1 in RabX2-2KO to ensure that complete knockout of one gene had not interfered with the expression of the adjacent gene product. RT-PCR results indicated that RabX2 mRNA expression in RabX1-2KO and RabX1 mRNA expression in RabX2-2KO was not detectably affected and found to be similar to the expression levels in wild type parasites (Figure 3C). We also performed immunofluorescence analysis to determine the locations of RabX1 or RabX2 proteins in the knockout cells. In RabX1-2KO, we were unable to detect any fluorescence corresponding to RabX1, but RabX2 was found localized at the Golgi complex (Figure 3D). Similarly, in RabX2-2KO we were able to detect RabX1 localized to reticular structures most probably representing the ER, while no fluorescence was detectable for RabX2 protein itself (Figure 3D). Thus, RabX1 and RabX2 were undetectable in their respective knockout cells and the complete knockout of one gene did not affect expression or localization of the neighboring gene product. In summary, Western blotting, Southern blotting, RT-PCR and immunofluorescence validates specific deletion of RabX1 and RabX2 in the respective knockout cells with independent effects on expression and location of the two proteins.

### RabX1 and RabX2 are non-essential in trypanosomes

**Figure 4:**
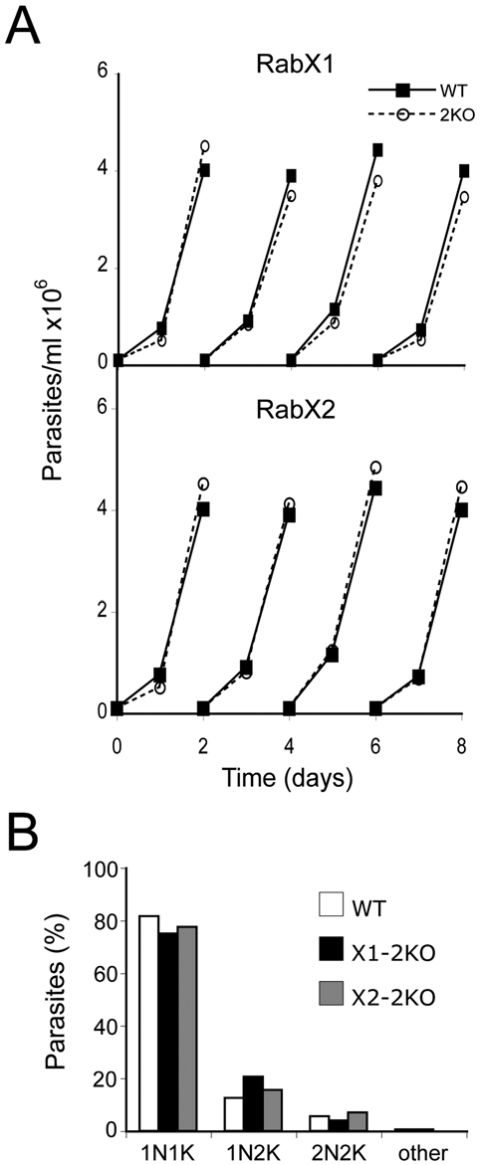
Knockout of neither RabX1 nor RabX2 has an effect on proliferation. (A) Growth curve for RabX1- and RabX2-2KO BSF mutants. RabX1- and RabX2-2KO have a similar growth rate to the wild type. Closed symbols, wild type; open symbols, knockout mutant. The analysis has been repeated twice (B). RabX1- and RabX2-2KO mutants have no defect in their cell cycle. RabX1- and RabX2-2KO mutant cells were cultured to 1×10^6^ cells/ml, fixed with 4% paraformaldehyde, adhered to poly-lysine slides and stained with DAPI. Their position in the cell cycle was determined by counting the number of nuclei and kinetoplasts in at least 200 individual cells.

While the successful generation of gene knockouts for RabX1 and RabX2 indicate that these genes are not essential, we investigated a possible role in normal proliferation of trypanosomes by analyzing the growth of knockout cells in *in vitro* culture. Growth curves were obtained by counting RabX1-2KO and RabX2-2KO cells every 24 hours over a period of eight days. Both RabX1- and RabX2-2KO parasites had no significant growth defect compared to the wild type parasites in culture (Figure 4A). The doubling times for wild type, RabX1-2KO and RabX2-2KO cells were 7 hours, 7.4 hours and 6.8 hours respectively, confirming no significant defect in their growth rate (Table 1). Examination of DAPI-stained parasites showed no significant changes to the distribution of parasites through the cell cycle (Figure 4B) and there was no appearance of parasites with abnormal nuclear or kinetoplast copy numbers, suggesting that RabX1 and RabX2 are not required for cell cycle progression. Hence neither RabX1 nor RabX2 are essential for normal cell cycle progression.

**Table 1:** Doubling times for wild type, RabX1 and RabX2 double knockout cell lines. Wildtype, RabX1-2KO and RabX2-2KO cells were grown at 37°C in HMI-9 media supplemented with 10% fetal calf serum. Cells were counted each day for up to eight days and doubling time calculated using the formula [(t_2_−t_1_) × {(log (2)/log (q_2_−q_1_)}]×24 where t_1_  =  initial time point, t_2_  =  final time point; q_1_  =  initial cell density and q_2_  =  final cell density.

Cell line	Average doubling time/hours (±sd)
WT	7.0 (±0.5)
RabX1-2KO	7.4 (±1.5)
RabX2-2KO	6.8 (±1.2)

**Figure 5:**
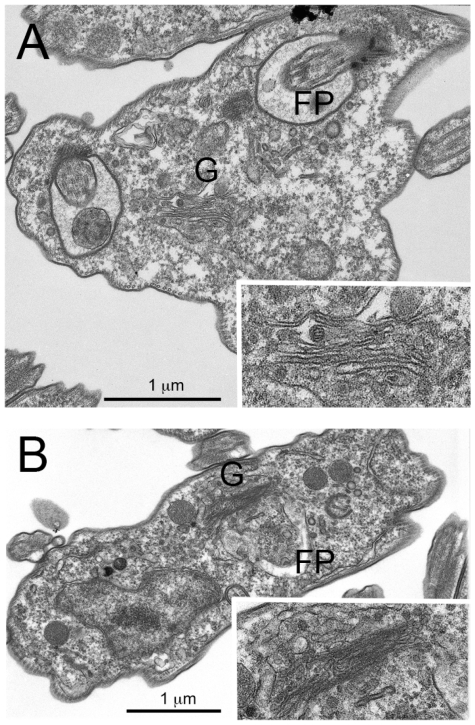
RabX1 and RabX2 are non-essential for normal endomembrane morphology. Ultrastructural analysis of RabX1- and RabX2-2KO mutants was performed using electron microscopy. (A) Electron micrograph of X1-2KO mutant showing no changes to the endomembrane compartments and the presence of unaltered ERGIC (inset). (B) Electron micrograph of X2-2KO mutant showing the presence of an unaltered Golgi apparatus (inset) with no changes to other endomembrane compartments. FP, flagellar pocket; G, Golgi apparatus.

Examination of the ultrastructure of RabX1- and RabX2-2KO parasites by electron microscopy revealed no major changes to the endomembrane architecture (Figure 5A and 5B). In RabX1-2KO, the structure of the ER was unaltered and the expected vesicular structures were seen associated with the Golgi complex, possibly the ERGIC (inset Figure 5A). Some material was observed within the flagellar pocket for both knockout cell lines; this may suggest defective lipid or other biosynthetic defects in these cells, but is also observed occasionally in wild type cultures. In RabX2-2KO, the structure of the Golgi complex is unaltered with the typical stacking pattern of the cisternae clearly visible (inset Figure 5B). Further, when GFP-tagged GRASP, a Golgi matrix protein, was expressed in the knockout cells it was found that the GFP-tagged protein was correctly localized to a single punctum in the correct position for the Golgi complex, suggesting no major structural abnormalities (Figure S1).

### RabX1 and RabX2 are non-redundant

To determine whether RabX1 and RabX2 act on the same pathway, we generated an RNAi construct to knockdown both RabX1 and RabX2 simultaneously. In the presence of tetracycline induction of double knockdown, we did not observe a growth defect suggesting that the two are non-redundant (Figure S2). Knockdown of RabX1 and RabX2 was confirmed by qRT-PCR with mRNA levels reduced by ∼70% and ∼50% respectively (Figure S2). Protein levels were also probed by Western immunoblotting and found to be knocked down by >60% for RabX1 and >50% for RabX2, consistent with the qRT-PCR data (Figure S2). While this level of suppression does not preclude the residual levels of RabX1 and RabX2 being sufficient for viability, the low levels of most Rab proteins normally makes them rather sensitive to knockdown, and hence these results potentially indicate that RabX1 and RabX2 are probably non-redundant, consistent with their differential location.

### Knockout of RabX1 or RabX2 has no effect on endomembrane system morphology

**Figure 6:**
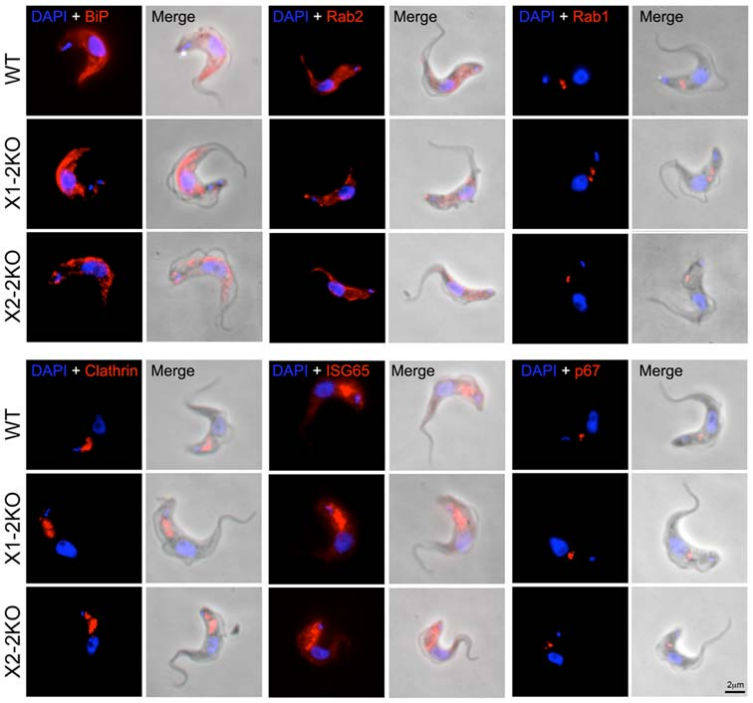
Knockout of RabX1 and RabX2 does not affect the location of endomembrane compartment markers. Immunofluorescence demonstrating the locations of BiP, clathrin, ISG65, p67, Rab1 and Rab2 (red) in both RabX1- and RabX2-2KO parasites. Parasites were counterstained with DAPI (blue) for DNA. Scale bar 2 µm. Note that the location of BiP and Rab2 with the ER, Rab1 with the Golgi complex, clathrin and ISG65 with endosomes and p67 with lysosomes is unchanged in both RabX1- and RabX2-2KO in comparison to wild type parasites. Multiple cells were analyzed and representative examples shown.

To investigate the effects of RabX1 or RabX2 knockout on the trafficking machinery, we first performed immunofluorescence analysis with established markers for various membrane-bound compartments. Immunofluorescence staining of cells with BiP or Rab2, both ER markers, indicated no changes to their localization at the ER in both RabX1-2KO or RabX2-2KO cells (Figure 6). Rab1, a Golgi complex marker, was normally localized in both the knockout lines indicating no changes to the morphology of the Golgi apparatus (Figure 6). Similarly, there was no change in the location of clathrin and ISG65, both endocytic markers, or p67, a lysosomal marker, in RabX1-2KO or RabX2-2KO cells (Figure 6). Thus the results from various immunofluorescence analyses indicate that RabX1 and RabX2 do not contribute to the structural integrity of the major compartments involved in exocytosis or endocytosis.

### RabX1 and RabX2 do not play a major role in endocytosis and exocytosis

**Figure 7:**
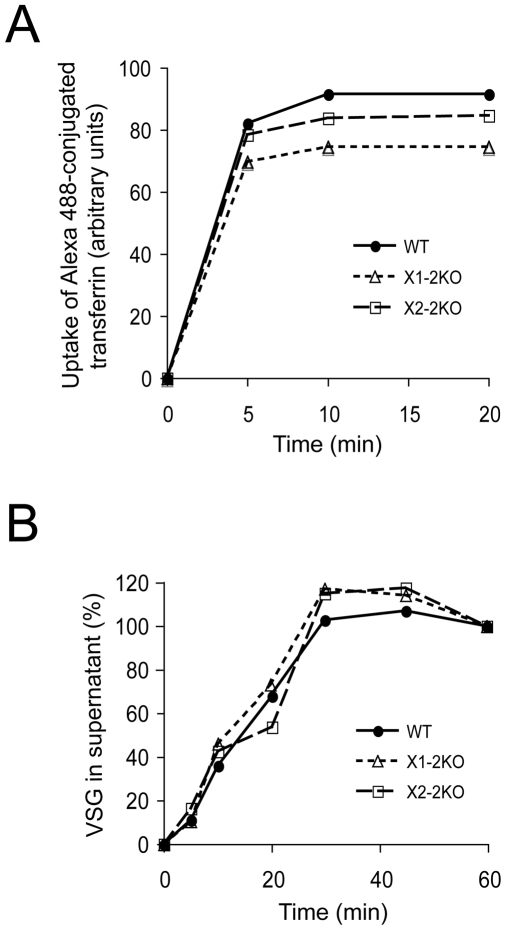
Endocytosis and exocytosis is not significantly altered in RabX1- and RabX2-2KO mutants. (A) Uptake of Alexa 488-conjugated transferrin in wild type, RabX1- and Rab X2-2KO mutants. Parasites grown to log phase were washed and incubated with Alexa 488-conjugated transferrin in their growth medium. Aliquots were taken at 0, 5, 10 and 20 minutes of incubation, cells washed to remove unbound transferrin and levels of intracellular transferrin determined by FACS. Uptake of Alexa 488-conjugated transferrin reached a maximum by 10 minutes in wild type, RabX1-2KO and RabX2-2KO parasites. Further, there was no significant difference in uptake between the wild type and the knockout lines. (B) Export of newly synthesized VSG in wild type, RabX1- and RabX2-2KO mutants. Parasites grown to log phase were pulse labeled with ^35^S-methionine. Samples were taken at 0, 20, 40 and 60 minutes of incubation and soluble VSG was hydrolyzed by GPI-PLC after hypotonic lysis of the parasites. Soluble VSG was quantified by analyzing VSG intensity using NIH ImageJ. Results are shown as percentage of total recovered soluble VSG after background subtraction. Note that there is no significant difference in the export of VSG by RabX1- and RabX2-2KO mutants compared to wild type parasites. Each analysis was performed twice with highly similar results.

To investigate the roles of RabX1 and RabX2 in endocytosis we analyzed the ability of the knockout cells to endocytose Alexa 488-conjugated transferrin, quantifying uptake by fluorescence-activated cell sorting (FACS). In wild type parasites transferrin accumulation reached a maximum by ten minutes (Figure 7A). Similarly RabX1-2KO cells and RabX2-2KO cells reached maximum uptake after ten minutes. There was also no major significant difference in the rate of transferrin uptake between the wild type and both of the knockout lines (Figure 7A). Both 2KO lines accumulated slightly less transferrin, and for the RabX2 knockout this was ∼20% less than wild type. However, the absence of any significant defect to uptake kinetics suggests that RabX1 and RabX2 do not play a major role in receptor-mediated endocytosis, and is consistent with the absence of any morphological effect on endocytic markers in the knockout cells and the absence of an enlarged flagellar pocket, both of which are associated with knockdown of early endocytic Rab proteins [42], [43].

The locations of RabX1 at the ER and RabX2 at the Golgi complex suggest a potential involvement in exocytosis, and the predominant cargo is newly synthesized VSG *en route* to the parasite surface. To determine if RabX1 and RabX2 are required for this process, we monitored the rate of transport of ^35^S-labeled VSG from the ER to the cell surface in both RabX1- and RabX2-2KO cells. In wild type and knockout cells the rate of VSG transport, as determined by the proportion of GPI-PLC-accessible VSG (sVSG) was highly similar (Figure 7B). This is consistent with our previous findings where RabX1 RNAi knockdown showed no effect VSG export [23]. These observations indicate that RabX1 and RabX2 are unlikely to play a major role in exocytosis of newly synthesized VSG. As VSG is by far the predominant product of the trypanosome ER, and no defects in ER or Golgi complex structure were detected in the knockout cell lines, these data suggest that RabX1 and RabX2 are most probably not required for exocytosis *per se*.

### RabX1 and RabX2 are non-essential for infection of mice

**Figure 8:**
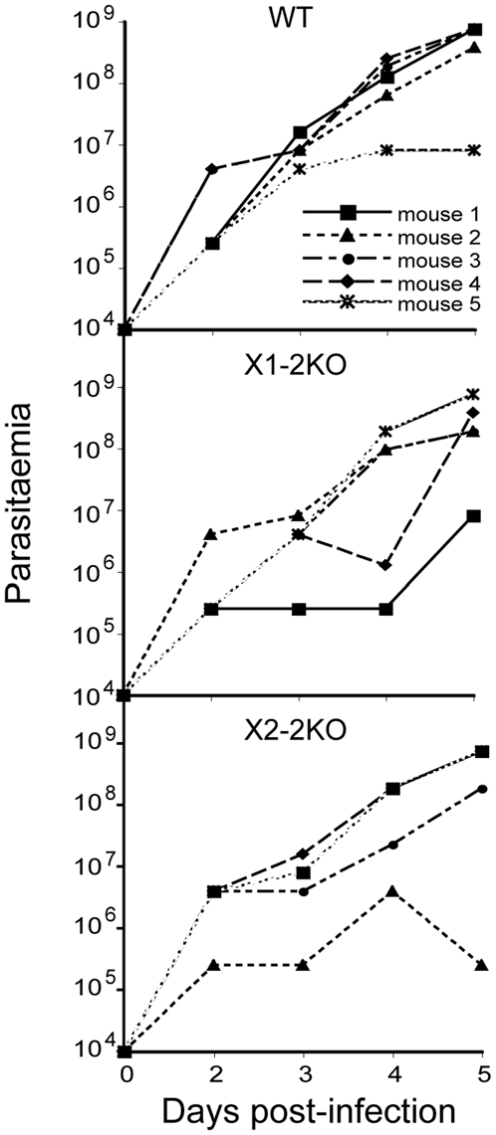
RabX1 and RabX2 are not required for mouse infectivity. 1×10^4^ wild type, RabX1- and RabX2-2KO parasites were used to infect five different mice. Parasites per ml of blood were recorded by tail bleeding from two to five days post-infection and parasitaemia levels from individual mice are shown. There was no significant difference in the ability of RabX1- and RabX2-2KO mutants to infect mice compared to the wild type parasites. Note that for RabX1- and RabX2-2KO infections similar levels of parasitaemia in individual mice have led to overlapping of the lines in the representative graph.

We next tested whether RabX1 and RabX2 have a role in infection or growth in the mammalian host by inoculating RabX1- and RabX2-2KO BSF from culture into mice. The knockout cell lines performed no differently to the wild type parasites, with a parasitaemia of ∼1×10^9^ parasites per ml typically produced by five days post-infection (Figure 8). These data suggest that RabX1 and RabX2 are not required for establishment of virulent infections in mice.

### RabX1 and RabX2 are involved in regulating infectivity in tsetse fly midgut

In preliminary experiments, wild type BSF 427 parasites, when fed to tsetse flies, were unable to establish infectivity as PCFs and instead were eliminated and disappeared from the tsetse midgut within 3 to 4 days; in contrast, both the RabX1-2KO and RabX2-2KO knockout lines showed some ability to survive and establish an infection in the insect. Moreover, while wild type cells failed to differentiate to PCF *in vitro*, the knockout lines differentiated to PCFs and proliferated for two weeks in culture.

**Figure 9:**
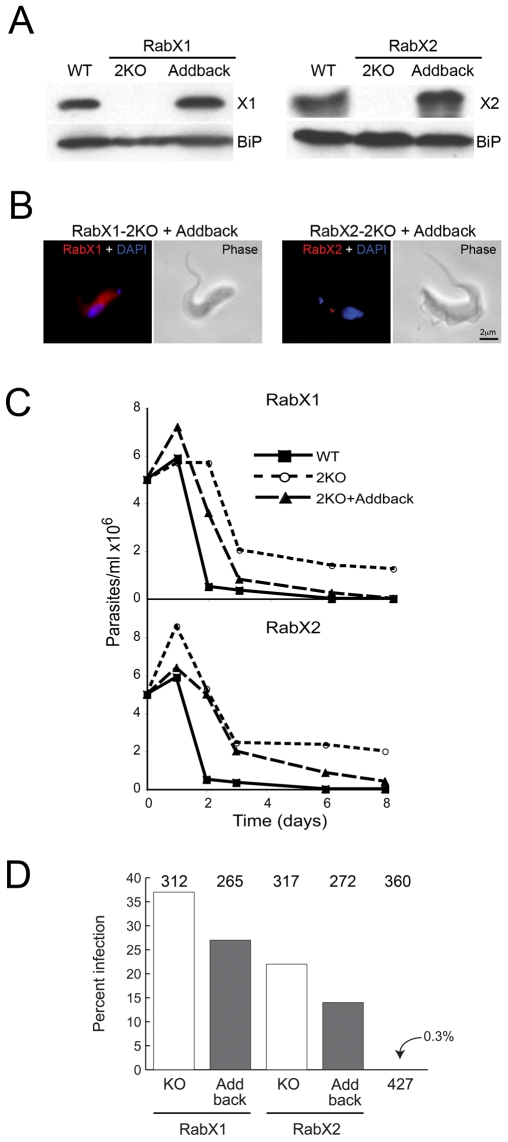
RabX1 and RabX2 play a role in differentiation of BSF to PCF. (A) Western blot analysis indicating over-expression of RabX1 and RabX2 in the add-back version of the respective knockout mutants. RabX1 and RabX2 are not detected in the respective 2KO mutants. BiP, an ER-marker was used as a loading control. (B) Immunofluorescence analysis showing the location of RabX1 to the ER and RabX2 to the Golgi apparatus in the add-back RabX1-2KO and RabX2-2KO cell lines respectively. Parasites were counterstained with DAPI (blue) for DNA. Phase-contrast images are shown adjacent to the respective fluorescence images. Scale bar 2 µm. (C) Proliferation of wild type, knockout mutants and the add-back lines of RabX1 and RabX2 during *in vitro* differentiation from BSF to PCF. BSF parasites grown to logarithmic phase were washed and incubated with SDM-79 medium at 5×10^6^ cells/ml and incubated at 27°C to induce differentiation to PCF. The number of parasites was counted up to eight days post-initiation of differentiation. Note that the knockout mutants were able to proliferate and maintain cell density more efficiently when compared to the wild type and the add-back lines. The experiment has been performed twice, with similar results. (D) Infection rates of wild type, knockout and add-back lines of RabX1 and RabX2 in tsetse flies dissected 4 to 5 days post-infection. Values at top indicate the numbers of flies dissected for the respective cell lines. The knockout mutants were able to infect tsetse midguts at significantly higher rates than wild type parasites (*P*<0.0001, Chi-squared). The add-back lines had lower infection rates that were significantly different from the knockout mutants (*P* = 0.02, Chi-squared).

To validate these observations we generated add-back cell lines, in which RabX1 and RabX2 was reintroduced into their respective knockout backgrounds as constructs targeted to the ribosomal spacer and under the control of the ribosomal RNA promoter. Positive clones were selected by culturing the transfected cells with hygromycin, neomycin and puromycin, and confirmed by Western blotting (Figure 9A). Both RabX1 and RabX2 add-back cells expressed the respective proteins at ∼two times greater than wild type levels (Figure 9A). Immunofluorescence analysis of the respective add-back cells indicates the location of RabX1 as ER and RabX2 as Golgi (Figure 9B). Neither add-back cell line exhibited a doubling time in *in vitro* culture that was significantly different to either the knockout or parental cells. Therefore Western blot and immunofluorescence indicates that reconstituted RabX1 and RabX2 are expressed and localized to the correct endomembrane compartment, *albeit* at slightly enhanced levels.

To further investigate the possible roles of RabX1 and RabX2 in BSF to PCF differentiation, we subjected the BSF wild type cells, the knockout lines and the add-back cell lines at 5×10^6^ parasites per milliliter to conditions favoring differentiation to PCF. Wild type parasites were able to continue proliferating as BSFs for about 24 hours but started dying afterwards and by six days post-induction there were no detectable live parasites (Figure 9C). However, both knockout cell lines were able to proliferate and, following a period of loss from the culture, were able to maintain a constant parasite number until eight days post-induction, and survived for up to 14 days (Figure 9C). The add-back cell lines had a near similar proliferation pattern to the wild type cells but retained a few live parasites until about day seven or eight (Figure 9C).

The ability of the knockout and reconstituted parasite cell lines to establish midgut infection in tsetse flies was assessed in flies dissected four to five days post-infection. Of 360 flies infected with the wild type, only a single trypanosome, resembling a long slender BSF in morphology and movement, was seen in one fly. However, both knockout cell lines showed 22–37% infected flies, an infection rate significantly different from the wild type (*P*<0.0001, Chi-squared) (Figure 9D). Additionally, the add-back cell lines gave significantly lower infection rates than for both X1-2KO (*P* = 0.01, Chi-squared) and X2-2KO (*P* = 0.02, Chi-squared) (Figure 9D). In summary, a major phenotypic distinction found between knockout cells and wild type is the enhanced ability of RabX1- and RabX2-2KO BSF to infect tsetse flies.

### Knockout of RabX1 and RabX2 does not lead to expression of differentiation markers

**Figure 10:**
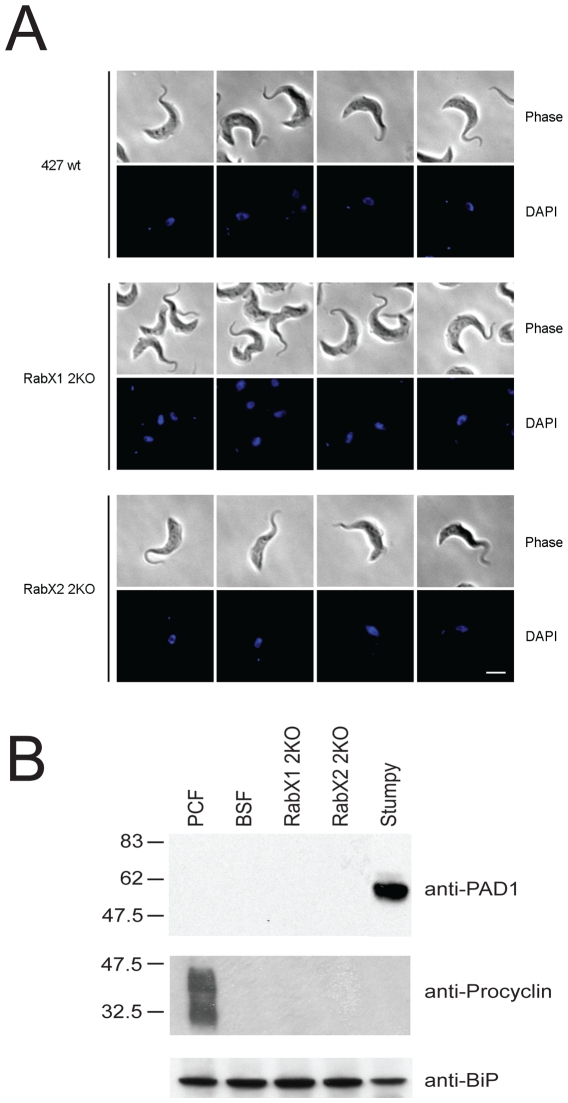
Stumpy and procyclic markers are not expressed in RabX1 or RabX2 gene knockouts. (A) Cell morphology comparison of knockout lines against wild type background cells. Cells were stained with DAPI and morphology was observed by phase contrast. Scale bar 2 µm. (B) Lysates from 427 PCFs, 427 BSFs, RabX1-2KO BSFs, RabX2-2KO BSFs and stumpy cells were probed with either anti-PAD1 or anti-procyclin antibodies. Molecular weight marker is shown on the left. BiP was used as loading control.

To determine whether RabX1-2KO and RabX2-2KO knockout lines are involved in established pathways for differentiation through the cell cycle, we checked for any morphological changes of the knockout lines versus the wild type 427 BSF. If a block to cell cycle progression was removed, we should witness the appearance of stumpy form cells. However, no observable change in cellular morphology was noted between wild type and knockout lines (Figure 10A), and is also consistent with the absence of obvious growth defects in the knockout cultures. This was confirmed by probing lysates from wild type 427 BSF, RabX1-2KO, RabX2-2KO, 427 PCFs and short stumpy cells with either antibodies against procyclin (PCF marker) or PAD1 (a stumpy-specific marker) [44]. We only detected procyclin in PCFs and PAD1 was exclusively detected in stumpy cells, suggesting that knockout of RabX1 or RabX2 did not lead to expression of either of these differentiation markers (Figure 10B).

## Discussion

There are sixteen members in the Rab subfamily of Ras-like small GTPases in *T. brucei*, within which RabX1, RabX2 and RabX3 are divergent at the sequence level from the fully canonical members, but remain on phylogenetic grounds within the greater Rab clade [18]. Here we addressed the evolutionary distribution and potential functions of RabX1 and RabX2 directly. While RabX1 and RabX2 are restricted to the trypanosomatids, we find evidence for expression in *T. brucei*, *T. cruzi* and *Leishmania,* suggesting a conserved and required role amongst a broad range of protists. This phylogenetic distribution, while uninformative in terms of deriving function based on studies in other taxa, does suggest a lineage-specific function, and prompts interest in these gene products. Previous studies from our laboratory demonstrated that RabX1 and RabX2 are specifically associated with exocytic endomembrane compartments, the ER and Golgi apparatus respectively, which was confirmed here. The only robust functional data previously gained are the appearance of ER-derived vesicles on over-expression of RabX1 and a minor endocytic defect for RabX2 [29], [41]. Further, RabX2 has undetectable GTPase activity in *in vitro* assays, suggesting that it is locked into the GTP-bound form [29]. However, we currently have no evidence to connect these factors with additional proteins or pathways, making extensive speculation concerning function unwarranted at this time. Using gene knockouts we obtained evidence indicating a potential role during the differentiation of bloodstream to procyclic forms for RabX1 and RabX2, but no major detectable function within intracellular transport.

Our experimental investigations of RabX1 and RabX2 functions were designed to address three specific questions. Firstly, is there evidence for a general growth or cell-cycle defect following suppression of the gene product or removal of the gene during *in vitro* culture? Secondly, is there evidence for effects on either the morphology or functioning of the endo/exocytic pathway? And, thirdly, what is the influence of RabX1 and RabX2 deletion on parasite growth *in vivo*, i.e. in the mammalian host and the tsetse insect vector?

RabX1 and RabX2 gene deletion in BSF cells or RNAi-mediated suppression in BSF and PCF cells were not associated with a proliferation defect in cultured parasites. In PCFs, RabX1 and RabX2 knockdowns are less potent, and hence it remains a possibility that RabX1 and RabX2 are required for efficient procyclic form proliferation but that a low level of expression is sufficient to facilitate this process. Further, using a chimeric RNAi construct to knockdown both RabX1 and RabX2 we found little impact on growth, suggesting that the absence of a growth defect for the single gene knockouts is unlikely due to redundancy between RabX1 and RabX2. This is also consistent with the fully distinct localizations of these two gene products.

Analysis of the ultrastructure of RabX1 and RabX2 knockout cells failed to reveal overt morphological changes to internal compartments, particularly the ER and Golgi complex, and this was corroborated by investigations of the localizations of a panel of marker proteins for intracellular compartments. In PCF parasites, over-expression of RabX1 leads to increased numbers of large vacuolar structures likely ER-derived [41] and over-expression of RabX2 results in a small increase to fluid-phase endocytosis [29], suggestive of a role in vesicle trafficking. In this study, which focused on the BSF, such defects were not found, suggesting either a developmental aspect to RabX1 and/or RabX2 function or that over-expression was able to reveal phenotypes not seen when gene product levels are decreased or eliminated. Using two assays designed to monitor important aspects of endocytosis and exocytosis, transferrin accumulation and VSG surface delivery respectively, we were unable to reveal a role for RabX1 or RabX2 in either process. As transferrin uptake is essential in BSF parasites [45] and represents one of the better characterized surface receptors [46], this evidence coupled with the absence of an enlarged flagellar pocket and normal clathrin location and expression levels, suggests no major role in endocytosis. Similarly, absence of an effect on VSG (this study and [23]), the major product of the trypanosome exocytic system, together with normal ER and Golgi apparatus locations and morphology, is also strong evidence against a major role in the anterograde exocytic pathway.

These findings were unexpected for two proteins that both localize to exocytic compartments and are part of the Rab family, *albeit* with some divergent sequence features. For example, RabX2 does not appear to have detectable GTPase activity indicating that it is constitutively GTP-bound [29]. This is likely due to an Ala-to-Ser substitution within the WD box which is known to result in loss of GTPase activity in human RhoE [28]. Furthermore, analysis of infectivity in mice indicated that neither RabX1 nor RabX2 were required for growth in the mammalian host; this latter finding was also unexpected as most other GTPases in trypanosomes are required for robust growth even *in vitro* [42], [43], [47], [48]. As infection of mice by cultured BSFs does not require a differentiation step, taken together these data could suggest that the major functions of RabX1 and RabX2 are outside of maintenance of general cell physiology and interactions with the mammalian host. Alternatively, as the 427 background strain is a rapidly growing and highly virulent strain, it is also possible that any requirement for RabX1 or RabX2 function in mouse infection has been lost. However, this is still consistent with the absence of a major and central role in virulence in the mammalian host. Initially, our results suggested that RabX1 and RabX2 might have a role during the differentiation between BSF and PCF, and in some manner participate in the transition between BSF and PCF. Most significantly, RabX1-2KO and RabX2-2KO knockout cells were able to establish infections within the tsetse midgut, in contrast to the parental cell line, and this phenotype was partially removed by complementation with RabX1 and RabX2, confirming specificity. The imperfect restoration of the parental behavior we attribute in the first instance to imprecise reexpression by RabX1 and RabX2 ectopic forms, which may also be due to removal of *in cis* flanking elements, known to influence expression levels in trypanosomes, and that could play a role in stage/differentiation specific expression. However, the inability to detect stage-specific markers for stumpy and procyclic stages in knockout cells questions whether RabX1 and RabX2 are truly involved in differentiation or just fly infectivity.

How might RabX1 and/or RabX2 contribute to a role in differentiation? Their precise roles remain unclear, while the apparent constitutive expression profile suggests that there may be more than one distinct role for these proteins, i.e. in different life stages. As RNA and/or protein levels are similar between the distinct *T. cruzi* and *T. brucei* life stages respectively [28], [29], [41], [49], this suggests a broadly constitutive expression profile, ruling out developmental expression as an aspect to function. Further, we considered the possibility that a transient change to RabX1 or RabX2 expression may occur during BSF to PCF differentiation, as reported previously from one of our laboratories for the RNA-binding TbZFP1, which participates in differentiation from BSF to PCF [49]. However, this was found not to be the case as both RabX1 and RabX2 protein levels remain constant during *in vitro* differentiation of a population of cells highly enriched in stumpy forms (Figure S3). Further, no obvious morphological change was observed in either knockout line, nor were either stumpy or procyclic markers induced in these cells as neither PAD1 nor procyclin were detected in BSF. Hence, despite a prolonged ability compared to the wildtype of the knockout cells to survive in tsetse flies we have no evidence connecting RabX1 or RabX2 to established differentiation pathways involving activation of the stumpy cell cycle arrest program and downstream events [49], [50].

The locations of RabX1 to the ER and RabX2 to the Golgi complex may suggest a possible role in remodeling of the ER and/or Golgi apparatus during fly infectivity. While we have no evidence to directly support this from the analysis presented here, and gross changes to exocytic activity appear unaffected in the BSF, it is known that the major antigens being processed through the exocytic system, expression of the major ER chaperone BiP, the activities of multiple glycosyltransferases and activity within the endocytic system are all under developmental regulation [12], [51], [52], [53], [54]. Hence, remodeling of the endomembrane system represents an important facet of the transition from one host to another or even between life stages within the same host, and one in which RabX1 and RabX2 could participate [2]. We cannot rule out other functions, for example in control of flagellar remodeling in transition to epimastigote or other stages. Detailed analysis of such events clearly requires examination of the effect of RabX1 and RabX2 knockout in trypanosome strains that are efficiently transmitted through tsetse.

The mechanisms underpinning the developmental shifts between life stages in trypanosomes are unclear, but more details have begun to emerge recently. Earlier work demonstrated the importance of temperature, Krebs cycle intermediates and cAMP in differentiation between BSF and PCF. More recently, evidence for the participation of RNA-binding proteins belonging to the ZFP family has been implicated, while a protein tyrosine phosphatase, TbPTP1, in its active form, has been reported to inhibit the differentiation of BSF to PCF [55]. How Ras-like GTPases contribute to this process is not known at present, and while there are a considerable number of candidate signaling GTPases encoded within the genome, the levels of sequence conservation are low and the predicted complexity of subtended signaling pathways rather simple, suggesting novel and/or divergent pathways are operating [19], [42]. The possibility that RabX1 and RabX2 act at some level to coordinate differentiation and intracellular trafficking is an attractive one, and the surprising absence of a major role in exocytosis or viability for differentiated cells either *in vitro* or *in vivo* is a further indication of how preliminary is the current level of understanding of G protein signaling in trypanosomatids.

## Supporting Information

Figure S1 (A) Western blot indicating over-expression of GRASP-GFP in RabX1-2KO and RabX2-2KO cells. GRASP-GFP is detected in the respective 2KO cells. BiP, an ER marker, was used as a loading control. (B) Indirect immunofluorescence showing the location of GRASP-GFP in RabX1-2KO and RabX2-2KO cells over-expressing GRASP-GFP. Parasites counterstained with DAPI (blue) for DNA. Phase contrast images are shown adjacent to the respective fluorescent images. Scale bar 2 µm.
(5.19 MB TIF)

Figure S2 RabX1 and RabX2 are non-redundant. (A) SMB cells transfected with p2T7-RabX1-RabX2 chimeric RNAi construct were grown in the presence (open squares) or absence (closed squares) of tetracycline for eight days. Growth curve shows a representative of an experiment performed in duplicate. No growth defect was observed. (B) RNAi knockdown of RabX1 and RabX2 mRNAs was confirmed by qRT-PCR after two days of tetracycline induction. Knockdown of RabX1 mRNA (∼70%) was more pronounced than for RabX2 mRNA (∼50%). (C) RabX1 and RabX2 proteins levels two days post-induction of RNAi knockdown were assessed by Western blotting with affinity-purified anti-RabX1 and anti-RabX2 antibodies. Graph shows quantitation of knockdown of RabX1 and RabX2 protein levels following normalization to BiP loading control. Knockdown for the chimera is similar for RabX1 and RabX2 as obtained with the individual ORF-targeted RNAi constructs.
(1.36 MB TIF)

Figure S3 Levels of RabX1 and RabX2 are unaltered during in vitro differentiation of T. brucei BSF to PCF. Trypanosomes containing >80% stumpy forms were placed under in vitro differentiation conditions and 1×107 cells were removed at 0, 2, 4, 6, 8, 10, 12 and 24 hours, boiled with SDS loading buffer, resolved on 12% SDS polyacrylamide gel and Western blotted.
(1.60 MB TIF)
