# Supplementary figures and images for: The Trypanosome Rab-Related Proteins RabX1 and RabX2 Play No Role in IntraCellular Trafficking but May Be Involved in Fly Infectivity
Source: PLoS One. 2009 Sep 29;4(9):e7217. doi: 10.1371/journal.pone.0007217 (PMC2748683; doi:10.1371/journal.pone.0007217)

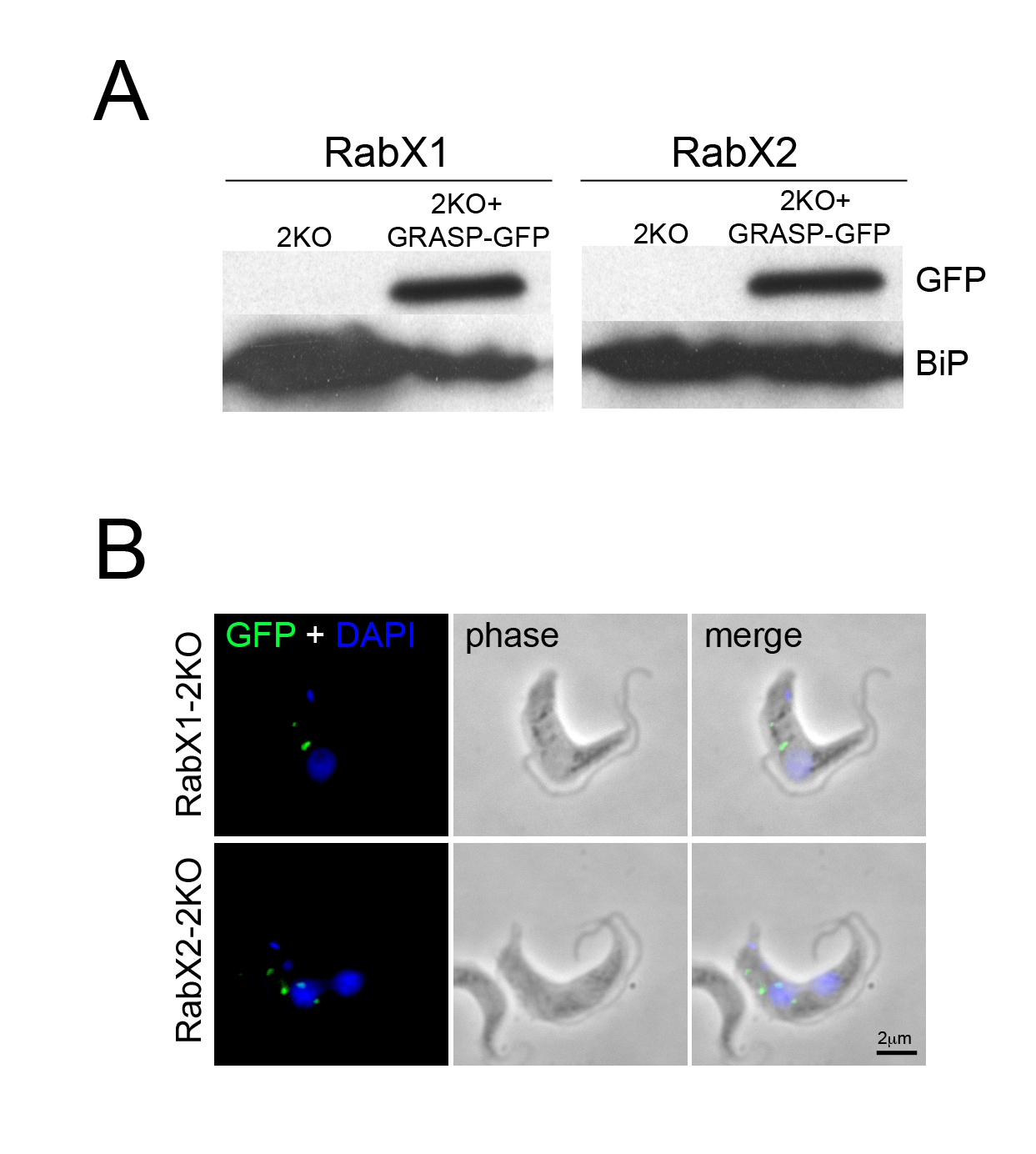

Supplement: Figure S1 — (A) Western blot indicating over-expression of GRASP-GFP in RabX1-2KO and RabX2-2KO cells. GRASP-GFP is detected in the respective 2KO cells. BiP, an ER marker, was used as a loading control. (B) Indirect immunofluorescence showing the location of GRASP-GFP in RabX1-2KO and RabX2-2KO cells over-expressing GRASP-GFP. Parasites counterstained with DAPI (blue) for DNA. Phase contrast images are shown adjacent to the respective fluorescent images. Scale bar 2 µm. (5.19 MB TIF) [file pone.0007217.s001.tif]

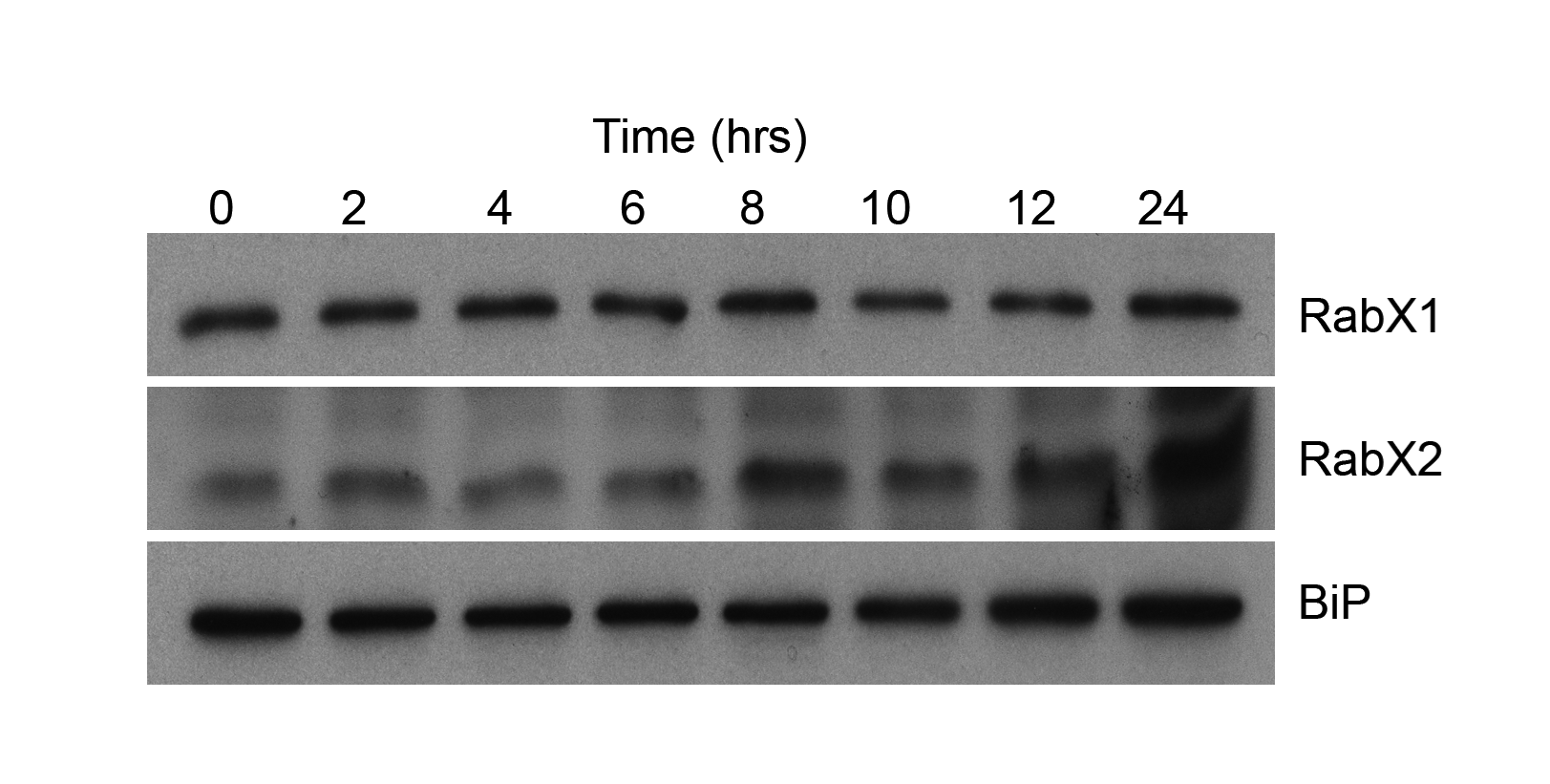

Supplement: Figure S2 — RabX1 and RabX2 are non-redundant. (A) SMB cells transfected with p2T7-RabX1-RabX2 chimeric RNAi construct were grown in the presence (open squares) or absence (closed squares) of tetracycline for eight days. Growth curve shows a representative of an experiment performed in duplicate. No growth defect was observed. (B) RNAi knockdown of RabX1 and RabX2 mRNAs was confirmed by qRT-PCR after two days of tetracycline induction. Knockdown of RabX1 mRNA (∼70%) was more pronounced than for RabX2 mRNA (∼50%). (C) RabX1 and RabX2 proteins levels two days post-induction of RNAi knockdown were assessed by Western blotting with affinity-purified anti-RabX1 and anti-RabX2 antibodies. Graph shows quantitation of knockdown of RabX1 and RabX2 protein levels following normalization to BiP loading control. Knockdown for the chimera is similar for RabX1 and RabX2 as obtained with the individual ORF-targeted RNAi constructs. (1.36 MB TIF) [file pone.0007217.s002.tif]

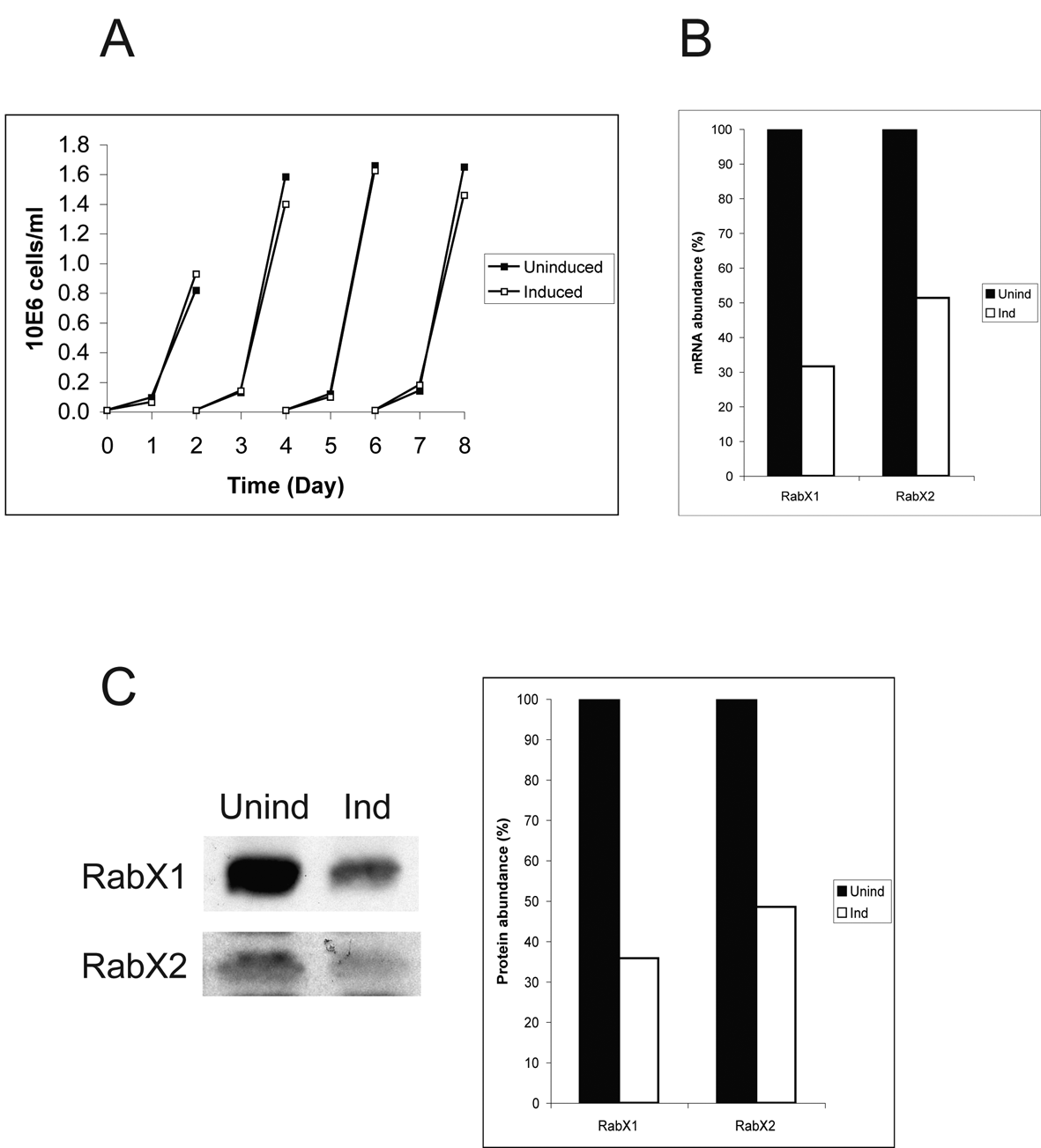

Supplement: Figure S3 — Levels of RabX1 and RabX2 are unaltered during in vitro differentiation of T. brucei BSF to PCF. Trypanosomes containing >80% stumpy forms were placed under in vitro differentiation conditions and 1×10^7^ cells were removed at 0, 2, 4, 6, 8, 10, 12 and 24 hours, boiled with SDS loading buffer, resolved on 12% SDS polyacrylamide gel and Western blotted. (1.60 MB TIF) [file pone.0007217.s003.tif]
